# Is There a Causal Link between Inflammation and Dementia?

**DOI:** 10.1155/2013/316495

**Published:** 2013-06-06

**Authors:** Ana-Maria Enciu, Bogdan O. Popescu

**Affiliations:** ^1^Department of Cellular and Molecular Medicine, School of Medicine, “Carol Davila” University of Medicine and Pharmacy, 8 Eroilor Sanitari, District 5, Bucharest 050474, Romania; ^2^Department of Neurology, Colentina Clinical Hospital (CDPC), School of Medicine, “Carol Davila” University of Medicine and Pharmacy, 19-21 Soseaua Stefan cel Mare, District 2, Bucharest 020125, Romania; ^3^Laboratory of Molecular Medicine, “Victor Babeş” National Institute of Pathology, 99-101 Splaiul Independenţei, District 5, Bucharest 050096, Romania

## Abstract

Neuroinflammation is a constant event in Alzheimer's disease (AD), but the current knowledge is insufficient to state whether inflammation is a cause, a promoter, or simply a secondary phenomenon in this inexorably progressive ailment. In the current paper, we review research data showing that inflammation is not a prerequisite for onset of dementia, and, although it may worsen the course of the disease, recent evidence shows that chronic inhibition of inflammatory pathways is not necessarily beneficial for patients. Prospective clinical trials with anti-inflammatory drugs failed to stop disease progression, measurements of inflammatory markers in serum and cerebrospinal fluid of patients yielded contradictory results, and recent bench research proved undoubtedly that neuroinflammation has a protective side as well. Knockout animal models for TNFRs or ILRs do not seem to prevent the pathology or the cognitive decline, but quite the contrary. In AD, the therapeutic intervention on inflammatory pathways still has a research future, but its targets probably need reevaluation.

## 1. Introduction

Based on current data, one cannot establish whether inflammation is a cause, a promoter, or simply a secondary phenomenon in Alzheimer's disease (AD) [1], although this can be the case for other molecular mechanisms involved in the pathogenic chain of events. In this paper, we shall try to argue that neuroinflammation, although indisputably present in AD, even as an early event, is not a prerequisite for onset of dementia—a syndrome that is met at a late stage of a disease (e.g., AD or vascular dementia (VaD) or B12 deficiency). Several clinical and laboratory research results support this perspective, and below we present three main arguments for this assumption.

*Argument No. 1.* The AD pathology (soluble A*β*/amyloid plaques) is one of the main triggers of neuroinflammation; increased levels of proinflammatory factors, such as cytokines and chemokines, and the activation of the complement cascade are known to occur in the brains of AD patients and to contribute to the local inflammatory response [2], but only after they are triggered by senile plaque. 

*Argument No. 2.* Neuroinflammation is a two-sided phenomenon, with both neurodestructive and neuroprotective facets; the hypothesis in which inflammation leads to dementia would imply a predominance of the first over the latter. Recent reports seem to indicate that, in AD brain, this is not the case, as showed further on in this review. 

*Argument No. 3.* Even though knockout animal models and anti-inflammatory treatment alleviate AD-like pathology in laboratory experiments, clinical trials were less successful, even contradictory, some reporting potentially hazardous adverse effects. Prospective clinical trials with anti-inflammatory drugs failed as trials with drugs designed for other targets—but it might be argued that inflammation is an early event and could be targeted for prevention [3].

In brief, our argumentation is presented in Table 1, which includes only a part of reported data in such an extensive research field.

## 2. Neuroinflammation-Related Events in AD

There are several definitions of inflammation available on http://medical-dictionary.thefreedictionary.com/inflammation, cropped from different medical dictionaries, all of them constructed around the central idea of “response to injury”.

Inflammatory response in the CNS occurs, similar to peripheral cascade of events, in few partially overlapping steps: (i) “classical” inflammation, leading to activation of macrophages/microglia and production of pro-inflammatory cytokines (TNF-*α*, IL-1*β*, and IL-12), chemokines, proteases, and redox proteins; (ii) alternative activation of microglia, a “repair/resolution” state, characterized by a switch in gene expression towards reconstruction and tissue repair, driven by IL-4 and IL-13 stimulation; and (iii) acquired deactivation, also an anti-inflammatory and repair functional phenotype, but this time induced by exposure of macrophages to apoptotic cells or to TGF-*β* and/or IL-10 [4]. From animal models of AD [5, 6] or histochemical analysis of human brain serial sections [7], one may conclude that this response is triggered by amyloid plaques, and a number of studies have reported aggregation of activated microglia around amyloid plaques in animal [5] and human brain [8–10]. Also soluble A*β* may be involved [11]. There are also reports of soluble A*β*-triggered neuroinflammation at BBB level [12], suggesting that inflammation is an early process in AD pathogeny. An increase in microglial activation has been observed in very early stages of AD and, interestingly, it is not preserved over time [13].

Inflammation-related gene profiling in AD patients brain samples indicates microglia to be in an alternative, “reconstructive” activation state, rather than the classical, “destructive” state, detrimental for neuronal homeostasis [14], making the anti-inflammatory therapeutic intervention questionable. In the view of neuroinflammation-driven AD pathology, there are numerous attempts to identify the signaling pathways that should be blocked in order to obtain effective therapeutic intervention. Although encouraging reports have been published following animal models experiments, later clinical study experiences showed that more likely a multimodal intervention is required, with emphasis on right timing rather than specific target.

Chronic blockade of IL-1R in a mouse model of AD alters brain inflammatory responses, alleviates cognitive deficits, markedly attenuates tau pathology, and partly reduces certain fibrillar and oligomeric forms of A*β* [15]. Unfortunately, brain-directed overexpression of human soluble IL-1 receptor antagonist in another transgenic mouse strain led to an atrophic phenotype of the brain along with modified levels of APP and PS1 [16]. It is possible, however, that such blockade stimulates alternative pathways, as reported by Reed-Geaghan et al. [17]. Although they reported decreased plaque burden and reduced levels of insoluble A*β* in an AD mouse model after CD14 deletion, loss of this TLR2/4 coreceptor expression was associated with increased expression of genes encoding the pro-inflammatory cytokines TNF-*α* and Ifn*γ*, decreased levels of the microglial/macrophage alternative activation markers Fizz1 and Ym1, and increased expression of the anti-inflammatory gene IL-10 [17]. 

Encouraging results were reported after deletion of the TNFR1 gene in APP23 transgenic mice, leading to diminished A*β* plaque formation, reduced beta-secretase 1 (BACE1) levels and activity, and overall unimpaired cognition [18]. However, treatment of AD patients with an anti-TNF*α* drug was unexpectedly poor in results, as discussed in a following section of this paper.

CNS expression of anti-inflammatory cytokine interleukin-4 attenuates Alzheimer's-disease-like pathogenesis in APP+PS1 double-transgenic mice, enhances neurogenesis, and inhibits spatial learning impairment [19]. Mechanisms proposed to accomplish these effects are activation of a subset of microglia and increased expression of A*β*-degrading enzymes [20]. AD patients chronically treated with acetylcholinesterase inhibitors have higher levels of serum IL-4 than nontreated subjects [21]; still, it is common knowledge that this kind of therapy does not prevent disease progression.

## 3. Neuroprotective Effect of ****Neuroinflammation 

In the complex environment of neuroinflammation, there is not a clear cut between “destructive” and “regenerative” actions of microglial subpopulations. The neuroprotective effects of activated microglia in inflammatory environments are partially overlapping its phagocytotic, destructive ones and may be triggered by signaling molecules produced by degenerating neurons, such as CX3C chemokine fractalkine (FKN). Secreted from damaged neurons, FKN promotes microglial phagocytosis of neuronal debris through the release of MFG-E8 and induces the expression of the antioxidant enzyme hemeoxygenase-1 (HO-1) in microglia, resulting in neuroprotection against glutamate toxicity [22].

Another such trigger is phosphatidylserine—a phospholipid expressed on the surface of apoptotic neurons—that, *in vitro*, induces a shift in microglia expression of cytokines from deleterious inflammatory (IL-1, TNF-*α*, NO) to protective (TGF-*β* and NGF) [23]. The deleterious effect of NO in AD was proven by Nathan et al. [24] in an animal model, in which deficiency of iNOS substantially protected the AD-like mice from premature mortality, cerebral plaque formation, increased A*β* levels, protein tyrosine nitration, astrocytosis, and microgliosis.

It has been proven that bone-marrow migrated macrophages are actively involved in brain A*β* clearance [25]. This process requires macrophage/microglial activation by chemokines and toll-like receptors. Knockout of CC chemokine receptor 2 (CCR2) in a mouse model of AD hastes the onset and worsens the cognitive impairment while compensatory increase in anti-inflammatory cytokines was noted [26]. Chronic exposure to CCR3 increased NMDA-evoked Ca(2+) signals in the hippocampal neurons and increased NMDA receptor levels [27]. Toll-like receptors (TLRs) are generally considered to activate pro-inflammatory pathways, and in the CNS they are upregulated in neuroinflammatory conditions such as multiple sclerosis (MS) [28] and its experimental animal model, experimental autoimmune encephalomyelitis (EAE) [29]. TLR 2 and 4 are reported to be present in amyloid plaques [30] and involved in uptake of A*β* and other aggregated proteins, thereby promoting their clearance from the CNS [31]. Another member of the family, TLR3, is induced on astrocytes in inflammatory conditions and, in turn, induces expression of a range of neuroprotective mediators, anti-inflammatory cytokines including IL-9, IL-10, and IL-11*. In vitro, *TLR3 activation enhances neuronal survival and endothelial cell growth, promoting neuroprotective responses rather than pro-inflammatory signals [32].

## 4. AD and MCI: Contradictory Data on CSF and Plasma Levels of Inflammatory Biomarkers

A meta-analysis of cytokines in serum and CSF of AD patients showed increased serum IL-6, TNF-*α*, IL-1*β*, TGF-*β*, and IL-12, whereas in CSF only TGF-*β* was increased as compared to control subjects [33]. Since CSF mirrors brain chemistry, this result can be interpreted as a confirmation of alternative, “repair” activation state of microglial population. Why TNF-*α* is not increased in the CSF, although it is a well-known mediator of inflammation and an inducer of apoptosis? It could be because in AD brains TNFRI levels are increased compared to nondemented brains. Furthermore, using 125I-TNF-*α* binding technique, Cheng et al. found that, in AD brains, 125I-TNF-*α* binding affinity to TNFRI is increased [34]; thus, the uptake of the ligand is increased and its disponibility in CSF is low. Low levels of TNF-*α* in frontal cortex, the superior temporal gyrus, and the entorhinal cortex compared with non-AD subjects have been previously reported [35]. However, in the same report, Lanzrein et al. describe no significant differences between AD subjects and controls regarding serum and CSF levels of interleukin-1 beta (IL-1*β*), IL-1 receptor antagonist (IL-1ra), interleukin-6 (IL-6), tumor necrosis factor-alpha (TNF-*α*), the soluble TNF receptors type I and II (sTNFR 1 and 2), and the acute phase protein alpha1-antichymotrypsin (*α*1-ACT). Diniz et al. also report no statistically significant differences in serum TNF-*α*, sTNFR1, and sTNFR2 between patients with MCI and AD as compared to controls. Nevertheless, patients with MCI who progressed to AD had significantly higher serum sTNFR1 levels as opposed to patients who retained the diagnosis of MCI upon followup [36]. Another study reports higher CSF levels of TACE/ADAM17 activity and soluble TNFRs in the MCI group than those in AD patients [37]. Buchhave et al. also report that patients with MCI who subsequently developed AD or VaD had higher levels of sTNFR1 and sTNFR2 in both CSF and plasma already at baseline when compared to age-matched controls [38]. An even more recent report found that TNF*α* expression was similar in AD and control brains [39].

Regarding inflammation and MCI progression to AD, there are reports to indicate the predictive value of certain pro-inflammatory cytokines for conversion. We already mentioned TNFR1, with several different groups of research in consensus, but also sCD40—another member of TNFR superfamily [40]—and IL-1*β*, but only for multidomain MCI, and not single-domain amnestic/nonamnestic MCI [41].

These data prove that there is an inflammatory process in MCI and AD, and it gets more intense as the disease advances. However, one cannot tell that the advancement is due to the inflammatory process, and recent reports for new markers for CSF diagnostic of AD may not even include any of the classical mediators of inflammation [42].

The panel of inflammatory cytokines and chemokines seems to be different in AD from other dementias, as MCP-1 is dramatically reduced in the grey matter in VaD and mixed dementia in comparison to controls, but not in AD. IL-6 decreases were also observed in the grey matter of VaD and mixed dementia [43]. Frontotemporal dementia (FTD) patients showed increased CSF TNF-*α* and TGF-*β* [44]. This kind of difference is not visible in the periphery, as already established AD and VaD cases had similar levels of TNF-*α* and IL-1*β* levels, when compared to each other and to healthy controls [45].

## 5. Inflammation-Mediators-Targeted Clinical Trials in AD

 As previously stated, inflammation is an important factor in AD pathogenesis; thus, the anti-inflammatory drugs were expected to have disease-modifying effects. Surprisingly, “classical” anti-inflammatory therapies failed to prevent dementia progression, as briefly discussed below.

### 5.1. Classical Therapies

Although both *steroidal and nonsteroidal anti-inflammatory* products were extensively tested, randomized clinical trials failed to demonstrate efficacy in AD [46]. Used for its antioxidant effects, *vitamin E*, alone [47] or in combination with other antioxidants (vitamin C, alpha-lipoic acid) [48], showed no improvement on cognition or CSF biomarkers of AD patients, but it raised questions regarding the safety of long-term administration [49, 50]. *Peroxisome proliferator-activated receptor-gamma agonists*, used for their anti-inflammatory effect (pioglitazone, rosiglitazone), provided contradictory data, with further need to assess safety issues [51]. *Omega-3 fatty acids* for 6 months did not influence inflammatory or biomarkers in CSF or plasma of patients with mild to moderate AD [52].

### 5.2. Molecular Therapies

*Etanercept, *an anti-TNF-*α* agent, seems to have a very narrow range of action, as it was reported to improve aphasia and verbal fluency in AD patients following CSF administration [53, 54]. 

*IL-R antagonists* are not yet used in clinical trials for neurodegenerative disorders, except for acute stroke [55] possibly because of putative beneficial role of neuroinflammation. A number of reports have provided evidence that activation of microglia and subsequent degradation of amyloid plaques may underlie this phenomenon (reviewed in [56]). 

In 2011, an oral inhibitor of receptor for advanced glycation end products (RAGE), PF-04494700, was tested for ten weeks in patients with mild to moderate AD and showed good tolerability and no serious adverse effects. However, no consistent effect on plasma levels of A*β*, inflammatory biomarkers, or secondary cognitive outcomes were reported [57].

## 6. Conclusions

Reports regarding pro- and anti-inflammatory cytokines in brains, serum, and CSF of AD patients are controversial. More useful in assessing the neuroinflammation role in cognitive decline and onset of dementia seems to be the study of inflammation markers in MCI patients. From the large panel of chemokines, cytokines, and cytokines receptors, CSF TNFR seem to correlate with progression from MCI to AD and show a rationale for anti-inflammatory treatment. Although CSF markers are intensively studied, they seem to be less useful as conversion predictors than neuropsychological and functional measures [58].

A reliable proof of inflammation as cause for dementia still lacks, and therefore its mandatory involvement in progression from MCI to AD remains questionable. However, inflammation is only one facet of the neuropathological profile of MCI, along with plaque and tangle formation, vascular pathologies, neurochemical deficits, cellular injury, oxidative stress, mitochondrial changes, changes in genomic activity, synaptic dysfunction, disturbed protein metabolism, and disrupted metabolic homeostasis [63].

## Figures and Tables

**Table 1:** Arguments for inflammation-driven onset of dementia.

	PROs	CONs
Cell cultures	A*β*-stimulated microglia produce and secret a number of proinflammatory molecules and neurotoxic factors [59] Pro-inflammatory cytokines attenuate microglial phagocytosis stimulated by fA*β* or complement receptor 3 [60]	IL-1*β* and TNF-*α* synergistically stimulate microglial NGF transcription release [61]

Animal models	IL-1R blockade alleviates cognitive deficits, markedly attenuates tau pathology, and partly reduces certain fibrillar and oligomeric forms of amyloid-*β* [15]	Prolonged central IL-1R blockade leads to a marked reduce in brain volume in transgenic mice [16]
	TNF-R1 deletion leads to diminished A*β* plaque formation, reduced beta-secretase 1 levels and activity, and overall unimpaired cognition [18]	3xTg-ADxTNF-RI/RII knockout mice exhibit enhanced amyloid and tau-related pathological features, due to reduced microglial-mediated uptake of extracellular amyloid-*β* peptide pools [62]

AD patients	MCI patients who later developed AD had higher serum levels of (i) TNFRI [36–38] (ii) soluble CD40 [40] (iii) IL-1*β* [41] than healthy controls AchE inhibitors increase serum IL-4 levels [21]	AD patients treated with etanercept showed some improvement, but did not match the animal models results Measurements of CSF/plasma cytokines are contradictory CSF TGF-*β* is increased in AD [33]—a putative effort for neurorepair It is common knowledge that AchE treatment only slows disease but does not stop or reverse its course

Abbreviations: IL: interleukin, TNF: tumor necrosis factor, NGF: nerve growth factor, CSF: cerebrospinal fluid, and AchE: acetylcholinesterase.
